# Assessing the Response of Ruminal Bacterial and Fungal Microbiota to Whole-Rumen Contents Exchange in Dairy Cows

**DOI:** 10.3389/fmicb.2021.665776

**Published:** 2021-06-01

**Authors:** Madison S. Cox, Courtney L. Deblois, Garret Suen

**Affiliations:** ^1^Department of Bacteriology, University of Wisconsin-Madison, Madison, WI, United States; ^2^Microbiology Doctoral Training Program, University of Wisconsin-Madison, Madison, WI, United States

**Keywords:** ruminal contents exchange, ruminal microbiota, milk production efficiency, dairy cattle, rumen

## Abstract

A major goal for the dairy industry is to improve overall milk production efficiency (MPE). With the advent of next-generation sequencing and advanced methods for characterizing microbial communities, efforts are underway to improve MPE by manipulating the rumen microbiome. Our previous work demonstrated that a near-total exchange of whole rumen contents between pairs of lactating Holstein dairy cows of disparate MPE resulted in a reversal of MPE status for ∼10 days: historically high-efficiency cows decreased in MPE, and historically low-efficiency cows increased in MPE. Importantly, this switch in MPE status was concomitant with a reversal in the ruminal bacterial microbiota, with the newly exchanged bacterial communities reverting to their pre-exchange state. However, this work did not include an in-depth analysis of the microbial community response or an interrogation of specific taxa correlating to production metrics. Here, we sought to better understand the response of rumen communities to this exchange protocol, including consideration of the rumen fungi. Rumen samples were collected from 8 days prior to, and 56 days following the exchange and were subjected to 16S rRNA and ITS amplicon sequencing to assess bacterial and fungal community composition, respectively. Our results show that the ruminal fungal community did not differ significantly between hosts of disparate efficiency prior to the exchange, and no change in community structure was observed over the time course. Correlation of microbial taxa to production metrics identified one fungal operational taxonomic unit (OTU) in the genus *Neocallimastix* that correlated positively to MPE, and several bacterial OTUs classified to the genus *Prevotella*. Within the *Prevotella*, *Prevotella_1* was found to be more abundant in high-efficiency cows whereas *Prevotella_7* was more abundant in low-efficiency cows. Overall, our results suggest that the rumen bacterial community is a primary microbial driver of host efficiency, that the ruminal fungi may not have as significant a role in MPE as previously thought, and that more work is needed to better understand the functional roles of specific ruminal microbial community members in modulating MPE.

## Introduction

Tremendous gains have been made in dairy cattle efficiency and productivity through efforts in breeding and management over the last several decades (Miglior et al., 2017). However, breeding for high production has resulted in consequent decreases in animal health and longevity (Knaus, 2009). This has led to an interest in breeding-independent strategies for improving milk production efficiency (MPE) to ensure a sustainable and economically viable future for the dairy industry. One promising avenue for improving MPE is through modulation of the rumen microbial community. The rumen microbiota is essential for the degradation of feed components into volatile fatty acids (VFAs), which have various fates once absorbed by the host. Inter-animal variability in rumen microbial metabolism can therefore result in differences in both milk volume and components by altering the pool of precursors available to the host. Importantly, the rumen microbial community has been repeatedly implicated in milk efficiency variability and production metrics in dairy cattle (Jami et al., 2014; Jewell et al., 2015; Shabat et al., 2016; Wallace et al., 2019).

The rumen contains a diverse, multi-domain microbial consortium of archaea, bacteria, fungi, and ciliate protozoa. Bacteria are the most abundant and diverse members of this community, with 10^10^–10^11^ cells/gram (Choudhury et al., 2015), and are the most thoroughly studied group in the rumen ecosystem. Fungi, which are much less abundant in the rumen at 10^3^–10^5^ cells/gram, are remarkably efficient fiber degraders that play an important role in the initial colonization and physical disruption of feed particles (Russell and Hespell, 1981; Akin and Borneman, 1990; Gordon and Phillips, 1998). Protozoa are known to have a role in VFA production, but in concert with archaea, are thought to have a greater importance in methane production, leading to a net energy loss.

The majority of studies that seek to implicate members of the rumen microbiome in host efficiency have focused primarily on the bacteria. However, due to the functional importance of the fungal community in digestion, recent consideration has been paid to the role of the ruminal fungi in overall fermentation (Puniya et al., 2015). For example, removal of anaerobic fungal populations in sheep has been demonstrated to negatively impact feed digestibility *in vivo* (Gao et al., 2013). Additionally, supplementation with rumen-derived fungal isolates increased feed digestibility and weight gain in buffalo calves (Tripathi et al., 2007). However, no studies to date have specifically linked native rumen fungal communities to performance metrics in dairy cattle.

Our previous study sought to provide evidence that near complete replacement of the rumen microbiota alone is sufficient to alter MPE (Weimer et al., 2017). In that study, an exchange of rumen contents was performed between three pairs of lactating Holstein cows with disparate MPE. For 7–10 days following the exchange, 5 of the 6 cows saw a reversal in MPE status from the pre-exchange baseline. This change in efficiency status was accompanied by a concurrent change in bacterial community structure. Within 10 days, MPE status and bacterial community structure returned to the expected baseline for the 5 affected hosts. This suggested a strong link between bacterial community composition and MPE, but also underscored the strong host-specificity of the adult rumen bacterial community, which apparently was able to re-establish even after extreme perturbation. We note that our previous study only included a cursory analysis of the ruminal bacterial community and did not consider other microbial community members such as the ruminal fungi.

Here, we expand on our previous findings by providing a more comprehensive analysis of the recovery of the rumen microbial community following near complete whole rumen contents exchange, including a focus on the ruminal fungal community. Given the demonstrated importance of the rumen fungal community in fiber degradation, we expect that high- and low-efficiency hosts would have distinct fungal communities prior to the exchange, and that the response of the rumen fungal community to the exchange protocol would mirror that of the bacterial community. Additionally, we sought to identify specific microbial community features, within both the bacteria and the fungi, that could be implicated in the observed differences in MPE.

## Materials and Methods

### Animal Trial and Sample Collection

The animal trial, DNA extraction, and bacterial community sequencing were performed as previously published in Weimer et al. (2017) under protocol A01427, as approved by the College of Agricultural and Life Sciences’ Animal Care and Use Committee, University of Wisconsin–Madison. Briefly, three pairs of healthy, lactating, ruminally cannulated 3rd-lactation Holstein dairy cows were selected on the basis of disparate dry matter intake (DMI) with similar energy corrected milk (ECM) over their first two lactation cycles, designated as either high or low milk production efficiency within each pair (HE and LE, respectively). An exchange of whole rumen contents was performed between the HE and LE member of each pair (∼95% of rumen contents removed), and ECM and DMI were recorded from 8 days prior to and 56 days following the exchange. Gross feed efficiency (GFE) was calculated as ECM/DMI. Solid- and liquid-phase rumen contents were collected at 18 timepoints relative to the exchange for characterization of microbial communities (sampling days relative to the exchange: −8, −7, −5, −4, −1, day 0 pre-exchange, day 0 post-exchange, 1, 2, 3, 7, 10, 14, 21, 28, 35, 42, 56).

### Amplicon Sequencing

Bacterial communities were characterized by sequencing of the variable 4 (V4) region of the 16S rRNA gene using a one-step protocol with barcoded primers (Kozich et al., 2013) and as previously described (Weimer et al., 2017). Fungal community sequencing was performed using custom barcoded primers designed according to the protocol outlined in Kozich et al. (2013). Barcodes were added to universal ITS4 primers (Taylor et al., 2016) (ITS4-Fun (5′-AATGATACGGCGACCACCGAGATCTACAC-TATGGTAATT-AA-AGCCTCCGCTTATTGATATGCTTAART-3′). Full sequences of barcoded primers can be found in Supplementary Data Sheet 1. A total of 50 ng of template DNA, 5 pmol of each primer, and 12.5 μL of 2X HotStart Ready Mix (KAPA Biosystems, Wilmington, MA, United States) and water to a total reaction volume of 25 μL were used for each PCR reaction. PCR conditions were: 3 min at 95°C for initial denaturation, 35 cycles of 30 s at 95°C, 30 s at 58°C, 30 s at 72°C, then 5 min at 72°C for final extension. Samples were run on a 1% low-melt agarose gel, and amplified DNA was extracted from the gel using the ZR-96 Zymoclean Gel DNA Recovery Kit (Zymo Research, Irvine, CA, United States). Extracts were equimolarly pooled and combined with the PhiX control library (Illumina, Inc., San Diego, CA, United States) at a 9:1 ratio. The combined library was loaded onto the Illumina MiSeq (Illumina, Inc., San Diego, CA, United States) for paired-end sequencing using the 2 × 300 bp v3 sequencing kit. Bacterial sequences from this project are deposited in the National Center for Biotechnology Information (NCBI) Short Read Archive (SRA) under the BioProject number PRJNA329260. Fungal sequences are deposited under BioProject number PRJNA695353.

### Sequence Cleanup

For both bacterial and fungal amplicons, sequences were demultiplexed by sample-specific indices on the Illumina MiSeq. Further processing and quality controls were performed in the program mothur v1.42.1 according to the most recent versions of our lab’s standard analysis pipelines (Supplementary Data Sheet 2), as adapted from the Schloss lab protocol (Kozich et al., 2013). Paired-end sequences were combined to form contigs and poor-quality contigs were removed from analysis. Bacterial sequences were aligned to the SILVA 16S rRNA gene reference alignment database v132 (Pruesse et al., 2007), and contigs that did not align to the V4 region were eliminated. Preclustering was performed (bacteria: diff = 2, fungi: diff = 4) to account for sequencing error, and fungal sequences were subjected to an internal Needleman alignment during this process (Needleman and Wunsch, 1970). Chimeric sequences were identified and removed using the UCHIME algorithm in mothur (Edgar et al., 2011). Sequences that could not be classified at the Kingdom level were eliminated. Singleton sequences were removed from the dataset prior to operational taxonomic unit (OTU) clustering.

### Sequence and Statistical Analysis

Clustering of OTUs at a sequence similarity of 97% was performed for both amplicons using the OptiClust algorithm in mothur (Westcott and Schloss, 2017). Bacterial sequences were classified using the SILVA 16S rRNA gene reference database v132 and fungal sequences were categorized using the UNITE v6.0 database (Nilsson et al., 2019), with a bootstrap cutoff of 80. Good’s coverage was calculated in mothur (Good, 1953). Normalization was applied in mothur (bacteria: 10,000 sequences/sample, fungi: 2,450 sequences/sample). Shannon’s Diversity (Shannon, 1948), Chao’s Richness (Chao, 1984), and post-normalization Good’s coverage were calculated in mothur. Representative sequences for each OTU were generated using the get.oturep command in mothur. The NCBI’s online nucleotide BLAST server was used to identify cultured isolates with high sequence similarity to representative sequences of select OTUs (Madden, 2002).

Statistical analysis was performed in R v3.6.3 (R Core Team, 2020) using RStudio v1.2.5033 (R Studio Team, 2020). Differences in alpha diversity statistics by time period and initial host efficiency status were assessed by two-way ANOVA, with Tukey’s HSD used as a *post hoc* test in the case of significance (*p* < 0.05). Non-significant interaction terms were removed from model formulae to better characterize main effects.

Beta diversity was calculated as Bray-Curtis dissimilarity (Bray and Curtis, 1957) and visualized with non-metric multidimensional scaling (NMDS) with square root transformed data in the R package vegan, v2.5-6 (Okansen et al., 2016). Differences in community structure between groups of samples were assessed by permutational multivariate ANOVA (vegan:adonis2, by = “margin”). Samples were assessed within sample type (solid or liquid fraction of the rumen contents) and amplicon (bacterial or fungal), with permutations stratified within individual to control for multiple sampling. Samples were assessed by initial efficiency status and categorical time within the sample period, as previously described (Weimer et al., 2017) (Pre = day -8 to day 0 pre-exchange, Post1 = day 0 post-exchange to day 7, Post2 = day 10 to day 56). These time periods were selected by Weimer et al. (2017) to capture the change in MPE status, which persisted for ∼7 days post-exchange, and the return to baseline MPE at 10–56 days post-exchange. Non-significant interaction terms were removed from model formulae to better characterize main effects. Pairwise comparisons between groups of samples were also calculated using the adonis function, with *P*-values FDR-corrected for multiple comparisons.

To streamline visualization of correlation networks, OTUs with <0.1% overall abundance were removed from the analysis (bacterial abundance cutoff: 2,146; fungal abundance cutoff: 522). Matrices of Pearson’s correlation coefficients were generated for within sample type, initial efficiency, and time period (Pre, Post1, Post2) using the rcorr function in the Hmisc package for R (Harrell Jr., 2020). Correlations that were weak (<0.70) or not highly significant (α < 0.001) were removed, and correlation matrices were used to generate correlation networks using igraph (Csardi and Nepusz, 2006). The degree-centrality of networks was calculated by domain using igraph. Differences in degree over time within initial efficiency status and domain were assessed using Kruskal-Wallis tests. Pairwise Wilcoxon tests were performed with FDR-correction applied to resultant *P*-values. Beanplots were generated using beanplot:beanplot in R (Kampstra, 2008). The 10 OTUs with the greatest degree centrality were selected from each of the pre-exchange networks (HE liquids, HE solids, LE liquids, and LE solids) and were subjected to Kruskal-Wallis testing to assess change over time, within sample type and host efficiency. *Post hoc* testing was performed as pairwise Wilcoxon rank sum tests with FDR correction applied to *P*-values. These OTUs were also correlated to production variables (ECM and GFE). Spearman’s ρ statistic was calculated between normalized OTU abundance and the phenotypic variable across all animals and time points, separated by sample type (liquids and solids). FDR correction was applied to *P*-values.

Individual species which differed between the categorical time periods were identified using the similarity percentages (SIMPER) function in vegan (vegan:simper) within amplicon, sample type, and initial efficiency status (Okansen et al., 2016). OTUs that explained >1% of the difference between time points were subjected to Kruskal-Wallis tests, with FDR-correction applied to resultant *P*-values.

Operational taxonomic units that were identified in the SIMPER analysis were correlated to phenotypic variables (ECM, GFE, molar fraction acetate, molar fraction propionate, molar fraction butyrate), as previously determined in Weimer et al. (2017). Spearman’s ρ statistic was calculated between OTU abundance and the variable of interest across all cows and time points, separated by amplicon (bacterial and fungal) and sample type. FDR-corrected *P*-values were calculated for each of these correlations using the cor.test function from the R package stats (R Core Team, 2020).

Linear discriminant analysis effect size (LEfSe) was performed on abundance-filtered, relative-abundance-transformed OTU matrices for Pre-exchange samples, within sample type and domain (Segata et al., 2011). The Huttenhower lab’s Galaxy instance was used with default parameters (Kruskal-Wallis *P* < 0.05, Pairwise Wilcoxon *P* < 0.05, logarithmic LDA score > 2.0)^1^ to obtain a list of candidate OTUs which were diagnostic of initial efficiency. Implicated OTUs were regressed against ECM and GFE, and *P*-values were FDR-corrected.

## Results

### Sequencing

Solid and liquid rumen samples were taken from three pairs of healthy lactating Holstein dairy cows at 18 timepoints over 64 days. These 216 samples were subjected to bacterial and fungal amplicon sequencing. Two samples were not subjected to fungal community sequencing because of insufficient DNA yields (RSL61d42 and RSL97d42). Bacterial sequencing generated 7,996,986 high-quality sequences with an average of 37,023 ± 2491 SE sequences per sample and a range of 10,049–328,315 sequences per sample. Fungal sequencing yielded 2,168,129 high-quality sequences an average of 10,131 ± 276 SE sequences per sample and a range of 2450–23,811 sequences per sample. Pre-normalization Good’s coverage was >97% for all bacterial samples, and >99% for all fungal samples, indicating that the sequencing depth was adequate to accurately characterize the communities of interest. Post-normalization there were a total of 2,147,014 bacterial and 523,784 fungal sequences used in analysis. Post-normalization Good’s coverage was >93% for all bacterial samples and >98% for all fungal samples. Sequencing results are summarized in Supplementary Data Sheet 3. Normalized OTU tables and taxonomic classification of OTUs can be found in Supplementary Data Sheet 4.

### Alpha Diversity

Alpha diversity analysis was performed for each amplicon and sample type separately (Supplementary Figure 1). In the liquid phase, changes in Shannon’s diversity for the bacterial community over the time course were not dependent on efficiency status (F_2_,_102_ = 1.679, *P* = 0.192). Ignoring efficiency status, there was no change in bacterial Shannon’s index by time period (F_2_,_104_ = 0.346, *P* = 0.708). Rumen liquids derived from HE animals had lower bacterial Shannon’s diversity than those derived from LE liquids (F_2_,_104_ = 18.353, *P* < 0.001). For the rumen solids, there was no significant interaction between efficiency status and time period for Shannon’s diversity of bacterial communities (F_2_,_102_ = 2.258, *P* = 0.110), and no significant difference between timepoints, irrespective of initial efficiency status (F_2_,_104_ = 0.359, *P* = 0.699). Similar to the liquid-derived samples, rumen solids derived from initially HE animals were less bacterially diverse than those derived from LE animals (F_2_,_104_ = 10.038, *P* = 0.002).

The impact of study period on Chao’s richness in liquid phase samples did not differ by initial efficiency status (F_2_,_102_ = 0.566, *P* = 0.569). Without consideration of efficiency, there was no change in bacterial species richness over time in the liquid samples (F_2_,_104_ = 1.023, *P* = 0.363). Overall, bacterial species richness in liquid samples did not differ by efficiency status (F_2_,_104_ = 1.228, *P* = 0.270). In solid-derived rumen samples, time period and efficiency status were not independent in their effect on Chao’s richness of bacterial communities (F_2_,_102_ = 3.883, *P* = 0.024). In these samples, HE cows had lower Chao’s richness than LE cows in the Post2 period (*P* = 0.037), and LE cows differed from Pre to Post2 (*P* = 0.025). All other pairwise comparisons were not significant (*P* > 0.05).

In the liquid phase, changes in fungal community Shannon’s diversity over time was independent of efficiency status (F_2_,_100_ = 2.069, *P* = 0.132). There was no change in liquid fungal community richness over time (F_2_,_102_ = 1.562, *P* = 0.215) or by efficiency status (F_2_,_102_ = 0.111, *P* = 0.739). In solid-phase samples, there was no significant interaction between study period and efficiency status (F_2_,_102_ = 0.111, *P* = 0.895). There was overall no change in richness by time period (F_2_,_104_ = 0.713, *P* = 0.493) or efficiency status (F_2_,_104_ = 0.410, *P* = 0.523).

The impact of time on fungal community richness liquid-phase samples was independent of efficiency status (F_2_,_100_ = 0.651, *P* = 0.524). There was no impact of study period on fungal community species richness in liquids (F_2_,_102_ = 1.423, *P* = 0.246), nor was there an impact of initial efficiency status on richness (F_2_,_102_ = 0.535, *P* = 0.466). Solid-phase fungal community richness likewise did not show an interaction between time period and efficiency status (F_2_,_102_ = 2.406, *P* = 0.095). There was no impact on fungal species richness in rumen solids-derived samples by either time period (F_2_,_104_ = 0.611, *P* = 0.545) or efficiency status (F_2_,_104_ = 0.304, *P* = 0.583).

### Beta Diversity

**FIGURE 1:**
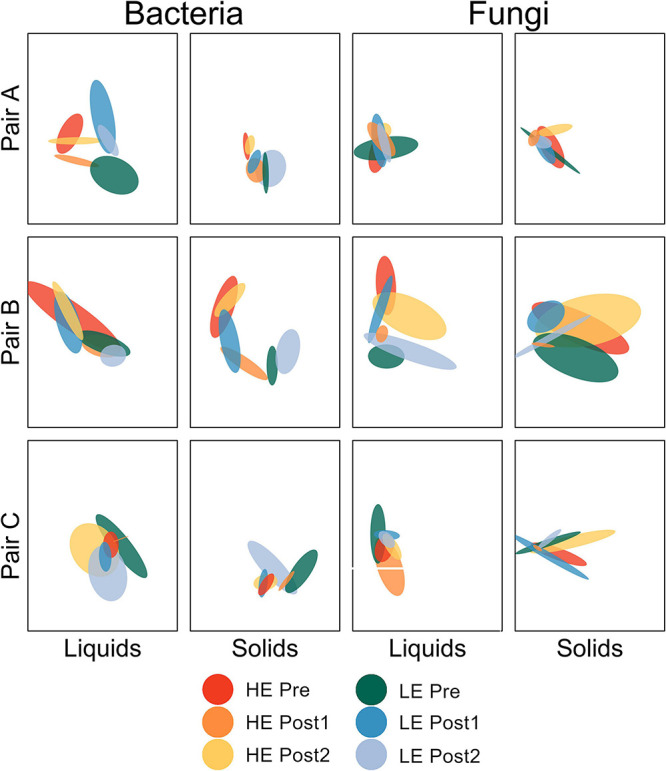
Non-metric multidimensional scaling plot depicting Bray-Curtis dissimilarity of bacterial and fungal communities by pair of host animals. Standard error ellipses are color coded by initial MPE of host and time within study.

Beta diversity analysis was performed for each amplicon and sample type separately. For ease of interpretation, pairs of animals were plotted separately (Figure 1). Between-sample diversity was calculated as Bray-Curtis dissimilarity and visualized with standard error ellipses to better illustrate the behavior of groups of points within a given host animal across the time series.

For the bacterial communities in the liquid phase (all three pairs of animals considered together), the change in community structure over time differed by efficiency status (*P* = 0.007). HE Pre was distinct from HE Post1 (*P* = 0.016), but not from HE Post2 (*P* = 1.000). LE Pre was not distinct from LE Post1 (*P* = 0.183) or LE Post2 (*P* = 0.708), but LE Post1 was distinct from LE Post2 (*P* = 0.016). All other pairwise comparisons were non-significant (*P* > 0.05). For the ruminal solids, the change in bacterial community structure over time was also dependent on efficiency status (*P* < 0.001). HE Pre was distinct from HE Post1 (*P* < 0.001), but only marginally distinct from HE Post2 (*P* = 0.090). HE Post1 was distinct from HE Post2 (*P* < 0.001). LE Pre was distinct from LE Post1 (*P* < 0.001), but not from LE Post2 (*P* = 0.178). LE Post1 was distinct from LE Post2 (*P* = 0.015). No other pairwise comparisons were significant (*P* > 0.05).

Fungal community structure change over time was dependent on efficiency status in rumen liquids (*P* = 0.012). HE Pre was not distinct from HE Post1 (*P* = 0.109) or HE Post2 (*P* = 0.510). HE Post1 was marginally distinct from HE Post2 (*P* = 0.054). LE Pre was marginally distinct from LE Post1 (*P* = 0.054), but not distinct from LE Post2 (*P* = 0.225). LE Post1 was distinct from LE Post2 (*P* = 0.044). All other pairwise comparisons were non-significant (*P* > 0.05). For the rumen solids, the impact of time period on fungal community structure differed by efficiency (*P* = 0.006). HE Pre was distinct from HE Post1 (*P* = 0.048), but not from HE Post2 (*P* = 338). HE Post1 was distinct from HE Post2 (*P* = 0.048). LE Pre was distinct from LE Post1 (*P* = 0.041), but not from LE Post2 (*P* = 0.114). LE Post1 was distinct from LE Post2 (*P* = 0.041). All other pairwise comparisons were non-significant (*P* > 0.05).

### Network Analysis

**FIGURE 2:**
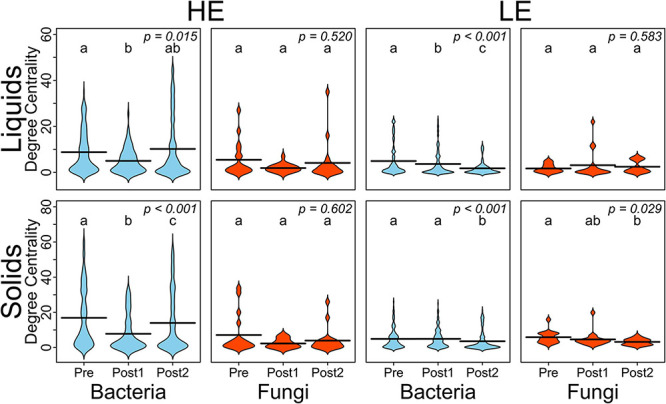
Beanplots expressing average degree-centrality of nodes by domain and over time for HE and LE-derived rumen liquids and solids. Shared letters indicate no difference in degree between timepoints (*P* > 0.05).

To better understand the interactions of the ruminal bacterial and fungal communities, as it relates to MPE, we conducted a correlation network analysis. Correlation networks were generated by time period (Pre, Post1, and Post2), separately for liquid and solids samples and for initial host efficiency (Supplementary Figure 2). Degree-centrality, which is the number of edges connected to a node in a network, was averaged within sample type, time point, host efficiency, and domain for each of the networks as summarized in Figure 2. In HE samples for both solid and liquid phases, the average degree-centrality of bacterial nodes in the network decreased from Pre to Post1, then recovered in Post2. This pattern was not upheld in LE samples, where the average bacterial node centrality either decreased and failed to recover (LE liquids) or did not decrease appreciably until Post2 (LE solids). The average degree-centrality of fungal nodes in these networks was largely unaffected by the exchange, except in the case of LE solids where there was a decrease from Pre to Post2.

The 10 nodes with the highest degree centrality scores were extracted from each of the four pre-exchange networks (HE Liquids, HE solids, LE Liquids, and LE Solids) and their variation over time was assessed (Table 1). Notably, many of the OTUs which increased significantly in Post1 in HE samples were classified to the genus *Prevotella_1* (B_OTU 4, B_OTU 5, B_OTU 6, B_OTU 10, B_OTU 24, B_OTU 54, B_OTU 70, and B_OTU 144). All of these OTUs also decreased in LE samples in this period, with the exception of B_OTU 6 and B_OTU 70, which did not change in these samples over the time course. Conversely, B_OTU 52, which classified to the genus *Prevotella_7*, increased in LE samples in Post1 and decreased in HE samples. One fungal genus was identified as a highly influential node in all four of the pre-exchange networks: F_OTU 3, which classified to the genus *Piromyces*. The abundance of this OTU increased in HE solids in the Post1 period but was unaffected in HE liquids and LE samples. The degree-implicated OTUs were also correlated to ECM and GFE, but no significant correlations resulted from this analysis (Supplementary Table 1).

**TABLE 1:** Kruskal-Wallis tests of influential OTUs in Pre-exchange networks.

**Block**	**OTU**	**Taxonomy**	**IN**	**Pre mean ± SE**	**Post1 mean ± SE**	**Post2 mean ± SE**	**χ^2^_*df=2*_**	***P*-value**
HE Liquids	B_OTU 5	Prevotellaceae;*Prevotella_1*	both	208.3 ± 33.0^*a*^	374.3 ± 36.5	235.2 ± 34.2^*a*^	9.669	0.020
	B_OTU 20	Lachnospiraceae;*Oribacterium*	both	72.0 ± 16.8^*a,b*^	19.0 ± 6.5^*a*^	92.2 ± 21.3^*b*^	9.540	0.020
	B_OTU 52	Prevotellaceae;*Prevotella_7*	both	115.7 ± 30.7^*a,b*^	44.3 ± 15.8^*a*^	132.9 ± 24.4^*b*^	5.870	0.078
	B_OTU 1	Succinivibrionaceae;*Succinivibrionaceae_UCG-001*	HE	1904.6 ± 301.4^*ab*^	1181.3 ± 362.6^*a*^	2153.0 ± 291.9^*b*^	6.314	0.067
	F_OTU 3	Neocallimastigaceae;*Piromyces*	HE	195.6 ± 27.0^*a*^	377.8 ± 36.8^*a*^	232.6 ± 25.4^*a*^	12.297	0.008
	B_OTU 4	Prevotellaceae;*Prevotella_1*	HE	122.1 ± 39.8^*a*^	200.1 ± 59.1	137.8 ± 50.2^*a*^	4.565	0.129
	B_OTU 7	Succinivibrionaceae;*Succinivibrionaceae_UCG-002*	HE	21.7 ± 7.3^*a*^	85.7 ± 16.8^*a*^	28.3 ± 8.7^*a*^	14.933	0.005
	B_OTU 24	Prevotellaceae;*Prevotella_1*	HE	22.6 ± 3.4^*a*^	48.5 ± 6.2	24.3 ± 4.3^*a*^	12.779	0.008
	B_OTU 54	Prevotellaceae;*Prevotella_1*	HE	8.1 ± 1.3^*a*^	9.7 ± 1.7	5.8 ± 0.9^*a*^	3.685	0.188
	B_OTU 65	Lachnospiraceae;Lachnospiraceae_XPB1014_group	HE	16.2 ± 4.1^*a*^	37.8 ± 4.0^*a*^	14.2 ± 3.6^*a*^	14.632	0.005
	B_OTU 70	Prevotellaceae;*Prevotella_1*	HE	3.8 ± 0.9^*a*^	6.7 ± 1.2	2.6 ± 0.7^*a*^	9.002	0.023
	B_OTU 131	Lachnospiraceae;Lachnospiraceae_XPB1014_group	HE	380.4 ± 73.6^*a,b*^	364.9 ± 43.8^*a*^	357.5 ± 70.2^*b*^	0.044	0.978
	B_OTU 6	Prevotellaceae;*Prevotella_1*	LE	153.0 ± 22.9^*a*^	268.1 ± 24.6	166.0 ± 20.2^*a*^	10.526	0.016
	B_OTU 27	Prevotellaceae;*Prevotella_7*	LE	220.6 ± 52.0^*a*^	129.3 ± 49.6^*a*^	220.5 ± 44.2^*a*^	1.483	0.503
	B_OTU 49	Veillonellaceae;Veillonellaceae_unclassified	LE	25.5 ± 5.5^*a,b*^	16.5 ± 5.7^*a*^	38.7 ± 6.6^*b*^	6.352	0.067
	B_OTU 81	Prevotellaceae;*Prevotella_1*	LE	23.4 ± 5.9^*a*^	9.0 ± 2.0^*a*^	21.8 ± 4.5^*a*^	5.299	0.096
	B_OTU 134	Lachnospiraceae_unclassified	LE	61.8 ± 26.7^*a*^	10.0 ± 4.7^*a*^	25.7 ± 10.1^*a*^	2.358	0.344
	B_OTU 168	Lachnospiraceae_unclassified	LE	9.9 ± 3.0^*a,b*^	5.1 ± 2.3^*a*^	13.5 ± 2.7^*b*^	6.455	0.067
	B_OTU 171	Prevotellaceae;Prevotellaceae_unclassified	LE	25.8 ± 5.3^*a*^	5.7 ± 2.3	27.0 ± 6.1^*a*^	14.313	0.005
HE Solids	B_OTU 24	Prevotellaceae;*Prevotella_1*	both	24.5 ± 7.7^*a*^	70.3 ± 14.2	27.7 ± 8.1^*a*^	11.713	0.020
	B_OTU 70	Prevotellaceae;*Prevotella_1*	both	10.4 ± 2.6^*a*^	19.4 ± 2.4	9.4 ± 1.7^*a*^	9.303	0.029
	B_OTU 131	Lachnospiraceae;Lachnospiraceae_XPB1014_group	both	7.6 ± 1.9^*a*^	16.8 ± 2.8	7.3 ± 1.5^*a*^	9.654	0.029
	B_OTU 17	Prevotellaceae;*Prevotella_1*	HE	72.2 ± 7.2^*a*^	77.6 ± 4.4^*a*^	73.6 ± 3.6^*a*^	0.894	0.639
	B_OTU 20	Lachnospiraceae;*Oribacterium*	HE	175.9 ± 35.0^*a,b*^	101.0 ± 39.9^*a*^	186.5 ± 29.2^*b*^	7.190	0.056
	B_OTU 35	Prevotellaceae;*Prevotella_7*	HE	103.6 ± 22.6^*a*^	38.7 ± 19.0^*a*^	75.4 ± 14.1^*a*^	4.590	0.121
	B_OTU 42	Ruminococcaceae_NK4A214_group	HE	41.7 ± 4.5^*a*^	54.2 ± 5.3^*a*^	42.0 ± 3.3^*a*^	5.606	0.083
	B_OTU 52	Prevotellaceae;*Prevotella_7*	HE	32.9 ± 7.5^*a*^	11.5 ± 4.3^*a*^	24.5 ± 4.1^*a*^	6.086	0.077
	B_OTU 83	Prevotellaceae;*Prevotella_1*	HE	14.3 ± 1.7^*a*^	9.2 ± 1.5^*a*^	9.3 ± 1.2^*a*^	5.469	0.083
	B_OTU 144	Prevotellaceae;*Prevotella_1*	HE	2.2 ± 0.5^*a*^	4.5 ± 0.7^*b*^	2.8 ± 0.5^*a,b*^	7.159	0.056
	F_OTU 3	Neocallimastigaceae;*Piromyces*	LE	107.2 ± 8.5^*a*^	150.4 ± 11.3	106.2 ± 5.9^*a*^	11.374	0.020
	B_OTU 10	Prevotellaceae;*Prevotella_1*	LE	60.0 ± 10.7^*a*^	53.3 ± 16.4	61.1 ± 7.3^*a*^	2.173	0.357
	B_OTU 49	Veillonellaceae;Veillonellaceae_unclassified	LE	25.3 ± 2.9^*a*^	35.5 ± 3.9^*a*^	25.1 ± 1.7^*a*^	5.945	0.077
	B_OTU 65	Lachnospiraceae_XPB1014_group	LE	8.6 ± 0.8^*a*^	5.4 ± 0.9^*a*^	7.3 ± 0.8^*a*^	5.981	0.077
	B_OTU 97	Bacteroidia_unclassified;Bacteroidia_unclassified	LE	10.4 ± 1.5^*a*^	24.8 ± 4.2^*a*^	12.3 ± 1.0^*a*^	13.470	0.020
	B_OTU 109	Lachnospiraceae;probable_genus_10	LE	24.4 ± 3.9^*a*^	24.7 ± 3.6	31.2 ± 2.7^*a*^	2.833	0.273
	B_OTU 1	Succinivibrionaceae;*Succinivibrionaceae_UCG-001*	HE	690.4 ± 205.6^*a*^	1106.7 ± 259.7^*a*^	422.1 ± 147.2^*a*^	6.464	0.068
	F_OTU 3	Neocallimastigaceae;*Piromyces*	HE	521.9 ± 58.9^*a*^	325.1 ± 82.2^*a*^	447.2 ± 68.9^*a*^	3.129	0.234
	B_OTU 4	Prevotellaceae;*Prevotella_1*	HE	359.6 ± 27.3^*a*^	261.3 ± 28.7	426.9 ± 36.7^*a*^	11.256	0.008
	B_OTU 7	Succinivibrionaceae;*Succinivibrionaceae_UCG-002*	HE	224.9 ± 30.1^*a*^	221.1 ± 79.5^*a*^	288.2 ± 64.4^*a*^	1.630	0.443
	B_OTU 24	Prevotellaceae;*Prevotella_1*	HE	72.6 ± 10.5^*a*^	31.7 ± 8.6	73.8 ± 8.5^*a*^	11.143	0.008
	B_OTU 54	Prevotellaceae;*Prevotella_1*	HE	53.4 ± 6.0^*a*^	31.7 ± 5.4	47.9 ± 3.2^*a*^	6.743	0.065
	B_OTU 65	Lachnospiraceae;Lachnospiraceae_XPB1014_group	HE	8.7 ± 1.2^*a*^	7.4 ± 1.3^*a*^	10.6 ± 1.1^*a*^	4.052	0.157
	B_OTU 70	Prevotellaceae;*Prevotella_1*	HE	29.8 ± 4.2^*a*^	20.8 ± 4.2^*a*^	30.6 ± 3.8^*a*^	2.115	0.367
	B_OTU 131	Lachnospiraceae;Lachnospiraceae_XPB1014_group	HE	7.2 ± 1.6^*a,b*^	3.9 ± 1.1^*a*^	8.0 ± 1.2^*b*^	5.656	0.080
	B_OTU 6	Prevotellaceae;*Prevotella_1*	LE	272.9 ± 21.4^*a*^	212.2 ± 25.7^*a*^	282.6 ± 18.9^*a*^	4.643	0.124
	B_OTU 27	Prevotellaceae;*Prevotella_7*	LE	37.5 ± 23.1^*a*^	103.1 ± 36.4^*a*^	33.0 ± 27.2	13.348	0.005
	B_OTU 49	Veillonellaceae;Veillonellaceae_unclassified	LE	9.9 ± 3.8^*a*^	20.0 ± 6.2^*a*^	7.0 ± 2.4^*a*^	5.854	0.080
	B_OTU 81	Prevotellaceae;*Prevotella_1*	LE	9.6 ± 3.4^*a*^	26.3 ± 5.6	10.1 ± 1.6^*a*^	12.639	0.005
	B_OTU 134	Lachnospiraceae_unclassified	LE	14.3 ± 10.0^*a*^	38.6 ± 21.7^*a*^	0.0 ± 0.0^*a*^	5.782	0.080
	B_OTU 168	Lachnospiraceae_unclassified	LE	2.0 ± 1.2^*a*^	6.5 ± 2.4	0.9 ± 0.5^*a*^	15.963	0.002
	B_OTU 171	Prevotellaceae;Prevotellaceae_unclassified	LE	5.1 ± 2.5^*a*^	30.2 ± 8.2	6.4 ± 1.8^*a*^	17.392	0.002
LESolids	B_OTU 24	Prevotellaceae;*Prevotella_1*	both	84.2 ± 9.4^*a*^	26.7 ± 7.3	66.6 ± 9.4^*a*^	15.590	0.002
	B_OTU 70	Prevotellaceae;*Prevotella_1*	both	22.7 ± 1.9^*a*^	10.3 ± 2.0	19.2 ± 2.2^*a*^	13.784	0.003
	B_OTU 131	Lachnospiraceae;Lachnospiraceae_XPB1014_group	both	26.3 ± 2.4^*a*^	18.6 ± 3.1^*a*^	24.5 ± 2.7^*a*^	4.449	0.150
	B_OTU 17	Prevotellaceae;*Prevotella_1*	HE	90.8 ± 5.4a	87.8 ± 4.1^*a*^	90.3 ± 6.2^*a*^	0.058	0.972
	B_OTU 20	Lachnospiraceae;*Oribacterium*	HE	9.8 ± 3.4^*a*^	110.4 ± 30.2	36.8 ± 24.2^*a*^	21.334	<0.001
	B_OTU 35	Prevotellaceae;*Prevotella_7*	HE	0.8 ± 0.3^*a*^	42.1 ± 12.9	13.7 ± 10.3^*a*^	11.099	0.010
	B_OTU 42	Ruminococcaceae_NK4A214_group	HE	62.1 ± 2.4^*a*^	41.4 ± 4.3	59.0 ± 3.4^*a*^	14.230	0.003
	B_OTU 52	Prevotellaceae;*Prevotella_7*	HE	1.2 ± 0.5^*a*^	15.9 ± 6.1	4.4 ± 2.6^*a*^	10.034	0.013
	B_OTU 83	Prevotellaceae;*Prevotella_1*	HE	6.6 ± 0.7^*a*^	9.9 ± 1.9^*a*^	6.4 ± 0.8^*a*^	2.083	0.424
	B_OTU 144	Prevotellaceae;*Prevotella_1*	HE	5.5 ± 0.6^*a*^	2.5 ± 0.5	5.3 ± 0.9^*a*^	15.398	0.002
	F_OTU 3	Neocallimastigaceae;*Piromyces*	LE	123.3 ± 23.8^*a*^	105.3 ± 26.6^*a*^	133.1 ± 23.2^*a*^	0.462	0.840
	B_OTU 10	Prevotellaceae;*Prevotella_1*	LE	170.4 ± 11.2^*a*^	128.7 ± 6.8^*b*^	146.9 ± 7.3^*a,b*^	9.451	0.015
	B_OTU 49	Veillonellaceae;Veillonellaceae_unclassified	LE	16.6 ± 4.7^*a*^	39.1 ± 8.0	15.0 ± 5.1^*a*^	10.450	0.012
	B_OTU 65	Lachnospiraceae_XPB1014_group	LE	47.1 ± 4.1^*a*^	41.9 ± 3.7^*a*^	43.4 ± 3.0^*a*^	0.820	0.747
	B_OTU 97	Bacteroidia_unclassified;Bacteroidia_unclassified	LE	5.3 ± 0.9^*a*^	7.3 ± 0.8^*a*^	5.0 ± 0.6^*a*^	4.444	0.150
	B_OTU 109	Lachnospiraceae;probable_genus_10	LE	31.4 ± 3.8^*a*^	18.7 ± 2.3	34.6 ± 3.9^*a*^	9.538	0.015
	B_OTU 110	Lachnospiraceae;Lachnospiraceae_unclassified	LE	13.3 ± 2.6^*a*^	18.5 ± 2.9^*a*^	14.9 ± 2.9^*a*^	2.329	0.401
	B_OTU 171	Prevotellaceae;Prevotellaceae_unclassified	LE	0.8 ± 0.2^*a*^	7.5 ± 1.2	2.9 ± 1.2^*a*^	25.028	<0.001

*The nodes with the 10 greatest values for degree centrality were selected. The implicating network is listed under “IN.” Tests were performed within blocks of sample type and efficiency status. *Post hoc* testing was performed as Wilcoxon rank sum tests with FDR correction applied to *P*-values. Means sharing letters are not significantly different (α = 0.05). All samples normalized to prior to analysis (bacteria: 10,000 sequences/sample, fungi: 2,450 sequences/sample).*

### Individual Taxa

We then sought to identify individual taxa within the bacterial and fungal communities that contributed to the observed phenotypic reversal of MPE by conducting a SIMPER analysis on two sets of contrasting time periods: Pre vs. Post1 and Post1 vs. Post2. This analysis was designed to identify taxa that were transferred into the new host from the donor, and that were present during the reversal of efficiency status, as previously described (Weimer et al., 2017). SIMPER-identified taxa that explained at least 1% of the variation in between any two time periods within efficiency status, rumen phase, and amplicon type were subjected to Kruskal-Wallis tests as summarized in Table 2.

**TABLE 2:** Kruskal-Wallis tests of SIMPER-implicated taxa.

**Amplicon**	**Testing Block**	**Implicating contrast(s)**	**OTU**	**Classification**	**Pre Mean ± SE**	**Post1 Mean ± SE**	**Post2 Mean ± SE**	**χ^2^_*df=2*_**	***P*-value**
Bacteria	HE Liquids	Pre/Post1	B_OTU 19	Prevotellaceae; *Prevotella_1*	55.6 ± 8.2^*a*^	139.7 ± 15.1	75.2 ± 10.4^*a*^	16.349	<0.001
		Pre/Post1, Post1/Post2	B_OTU 3	Prevotellaceae; *Prevotella_1*	322.3 ± 27.3^*a*^	414.3 ± 29.4	320.2 ± 20.5^*a*^	6.953	0.031
			B_OTU 4	Prevotellaceae; *Prevotella_1*	195.6 ± 27.0^*a*^	377.8 ± 36.8	232.6 ± 25.4^*a*^	12.297	0.002
			B_OTU 5	Prevotellaceae; *Prevotella_1*	208.3 ± 33.0^*a*^	374.3 ± 36.5	235.2 ± 34.2^*a*^	9.669	0.007
			B_OTU 6	Prevotellaceae; *Prevotella_1*	153.0 ± 22.9^*a*^	268.1 ± 24.6	166.0 ± 20.2^*a*^	10.526	0.005
		Post1/Post2	B_OTU 1	Succinivibrionaceae; *Succinivibrionaceae UCG-001*	1904.6 ± 301.4^*a,b*^	1181.3 ± 362.6^*a*^	2153.0 ± 291.9^*b*^	6.314	0.043
			B_OTU 2	Prevotellaceae; *Prevotella_1*	653.5 ± 77.8^*a*^	759.8 ± 53.9^*a*^	593.2 ± 59.9^*a*^	5.012	0.082
			B_OTU 29	Prevotellaceae; *Prevotella_7*	145.7 ± 33.6^*a*^	119.6 ± 55.8^*a*^	251.1 ± 46.7^*a*^	7.214	0.027
			B_OTU 52	Prevotellaceae; *Prevotella_7*	115.7 ± 30.7^*a,b*^	44.3 ± 15.8^*a*^	132.9 ± 24.4^*b*^	5.870	0.053
	HE Solids	Pre/Post1, Post1/Post2	B_OTU 18	Lachnospiraceae; *Butyrivibrio_2*	61.4 ± 8.6^*a*^	124.0 ± 19.7	62.5 ± 6.6^*a*^	10.512	0.005
		Post1/Post2	B_OTU 7	Succinivibrionaceae; *Succinivibrionaceae UCG-002*	56.3 ± 19.2^*a*^	151.5 ± 35.0	62.2 ± 21.9^*a*^	7.181	0.028
			B_OTU 20	Lachnospiraceae; *Oribacterium*	175.9 ± 35.0^*a,b*^	101.0 ± 39.9^*a*^	186.5 ± 29.2^*b*^	7.190	0.027
	LE Liquids	Post1/Post2	B_OTU 1	Succinivibrionaceae; *Succinivibrionaceae UCG-001*	690.4 ± 205.6^*a,b*^	1106.7 ± 259.7^*a*^	422.1 ± 147.2^*b*^	6.464	0.039
			B_OTU 3	Prevotellaceae; *Prevotella_1*	423.9 ± 26.3^*a,b*^	367.7 ± 23.5^*a*^	462.8 ± 27.6^*b*^	6.930	0.031
			B_OTU 4	Prevotellaceae; *Prevotella_1*	359.6 ± 27.3^*a*^	261.3 ± 28.7	426.9 ± 36.7^*a*^	11.256	0.004
			B_OTU 5	Prevotellaceae; *Prevotella_1*	398.8 ± 33.1^*a*^	278.2 ± 35.1	462.3 ± 31.8^*a*^	12.875	0.002
			B_OTU 27	Prevotellaceae; *Prevotella_7*	37.5 ± 23.1^*a*^	103.1 ± 36.4^*a*^	33.0 ± 27.2	13.348	0.001
			B_OTU 29	Prevotellaceae; *Prevotella_7*	38.1 ± 23.8^*a*^	112.0 ± 44.5	47.0 ± 35.3^*a*^	12.078	0.002
			B_OTU 52	Prevotellaceae; *Prevotella_7*	33.9 ± 21.4^*a*^	74.8 ± 26.6	20.2 ± 15.4^*a*^	14.165	<0.001
	LE Solids	Pre/Post1	B_OTU 5	Prevotellaceae; *Prevotella_1*	153.8 ± 20.7	79.4 ± 13.4^*a*^	100.7 ± 14.6^*a*^	9.063	0.011
		Pre/Post1, Post1/Post2	B_OTU 20	Lachnospiraceae; *Oribacterium*	9.8 ± 3.4^*a*^	110.4 ± 30.2	36.8 ± 24.2^*a*^	21.334	<0.001
Fungi	HE Liquids	Pre/Post1	F_OTU 5	Neocallimastigaceae; *Neocallimastix*	141.2 ± 22.5^*a*^	61.5 ± 8.3^*b*^	117.0 ± 23.0^*a,b*^	9.305	0.010
		Pre/Post1, Post1/Post2	F_OTU 7	Saccharomycetales Incertae sedis; *Wickerhamomyces anomalus*	149.1 ± 29.7^*a*^	45.0 ± 19.3	170.0 ± 48.4^*a*^	14.854	<0.001
		Post1/Post2	F_OTU 2	Neocallimastigaceae; unclassified	277.9 ± 46.2^*a*^	513.8 ± 74.2	239.4 ± 38.9^*a*^	9.678	0.008
			F_OTU 9	Neocallimastigaceae; *Piromyces*	44.6 ± 13.2^*a,b*^	44.3 ± 7.5^*a*^	20.1 ± 3.8^*b*^	6.436	0.040
	HE Solids	Pre/Post1	F_OTU 5	Neocallimastigaceae; *Neocallimastix*	209.9 ± 28.4	75.3 ± 7.9^*a*^	111.3 ± 17.3^*a*^	20.020	<0.001
			F_OTU 6	Neocallimastigaceae; *Neocallimastix*	88.1 ± 14.3^*a*^	145.5 ± 8.1^*b*^	121.0 ± 13.8^*a,b*^	7.192	0.027
			F_OTU 10	Saccharomycetales Incertae sedis; *Debaryomyces prosopidis*	64.2 ± 36.7^*a*^	5.1 ± 3.3	15.6 ± 5.1^*a*^	9.556	0.008
		Pre/Post1, Post1/Post2	F_OTU 2	Neocallimastigaceae; unclassified	310.5 ± 62.8^*a*^	518.9 ± 52.4	242.6 ± 60.8^*a*^	10.504	0.005
			F_OTU 3	Neocallimastigaceae; *Piromyces*	67.5 ± 23.0^*a*^	92.3 ± 13.8	41.1 ± 13.1^*a*^	9.950	0.007
			F_OTU 7	Saccharomycetales Incertae sedis; *Wickerhamomyces anomalus*	141.1 ± 68.6^*a*^	7.6 ± 1.9	238.4 ± 84.9^*a*^	18.930	<0.001
		Post1/Post2	F_OTU 4	Neocallimastigaceae; *Piromyces*	182.6 ± 28.7^*a,b*^	186.9 ± 14.9^*a*^	110.4 ± 22.7^*b*^	7.749	0.021
			F_OTU 11	Trichocomaceae; *Penicillium roqueforti*	2.9 ± 1.6^*a*^	0.3 ± 0.2^*a*^	173.0 ± 81.7	32.528	<0.001
	LE Liquids	Pre/Post1, Post1/Post2	F_OTU 5	Neocallimastigaceae; *Neocallimastix*	62.4 ± 23.5^*a*^	158.2 ± 21.0^*b*^	71.3 ± 8.4^*c*^	19.725	<0.001
		Post1/Post2	F_OTU 4	Neocallimastigaceae; *Piromyces*	323.0 ± 31.6^*a*^	387.4 ± 68.3^*a*^	147.2 ± 16.9	19.223	<0.001
			F_OTU 7	Saccharomycetales Incertae sedis; *Wickerhamomyces anomalus*	54.8 ± 11.4^*a*^	24.2 ± 5.4	105.2 ± 26.3^*a*^	9.436	0.009
			F_OTU 11	Trichocomaceae; *Penicillium roqueforti*	1.3 ± 0.7^*a*^	0.1 ± 0.1^*a*^	44.0 ± 15.6	31.621	<0.001
	LE Solids	Pre/Post1	F_OTU 5	Neocallimastigaceae; *Neocallimastix*	64.3 ± 8.7^*a*^	199.7 ± 26.8^*b*^	120.6 ± 11.7^*c*^	22.600	<0.001
		Pre/Post1, Post1/Post2	F_OTU 7	Saccharomycetales Incertae sedis; *Wickerhamomyces anomalus*	37.6 ± 12.7^*a*^	8.7 ± 3.8	32.4 ± 13.6^*a*^	11.572	0.003
		Post1/Post2	F_OTU 4	Neocallimastigaceae; *Piromyces*	252.2 ± 36.4^*a,b*^	298.1 ± 46.2^*a*^	168.6 ± 18.3^*b*^	7.687	0.021
			F_OTU 11	Trichocomaceae; *Penicillium roqueforti*	1.0 ± 0.3^*a*^	0.1 ± 0.1^*b*^	112.0 ± 96.4^*c*^	26.006	<0.001

*Tests performed within amplicon, sample type, and efficiency status. *Post hoc* testing was performed as Wilcoxon rank sum tests with FDR correction applied to *P*-values. Means sharing letters are not significantly different (α = 0.05). All samples normalized to prior to analysis (bacteria: 10,000 sequences/sample, fungi: 2,450 sequences/sample).*

Similar to the OTUs implicated in the above network analysis, many of the bacterial OTUs in the liquid phase derived from HE cows that increased significantly in Post1, relative to Pre and Post2, were classified to the genus *Prevotella_1* (B_OTU 3, B_OTU 4,B_OTU 5, B_OTU 6, and B_OTU 19). In addition, many OTUs that tended to decrease in Post1 were classified to *Prevotella_7* (B_OTU 29 and B_OTU 52). The opposite pattern was observed in LE liquid samples: OTUs classifying to *Prevotella_1* tended to decrease in Post1 relative to Pre and Post2 (B_OTU 3, B_OTU 4, and B_OTU 5), and those classifying to *Prevotella_7* tended to increase (B_OTU 29 and B_OTU 52). In solid-derived samples from HE animals, an OTU classifying to the genus *Oribacterium* (B_OTU 20) was more abundant in Post2 than Post1, though neither differed from Pre. This OTU showed a marked increase in Post1 in LE solids, then returned to baseline abundance in Post2. An OTU classified to Succinivibrionaceae *UCG_002* (B_OTU 7) and another classified to *Butyrivibrio_2* (B_OTU 18) were more abundant in HE solid samples in Post1 relative to Pre and Post2.

**FIGURE 3:**
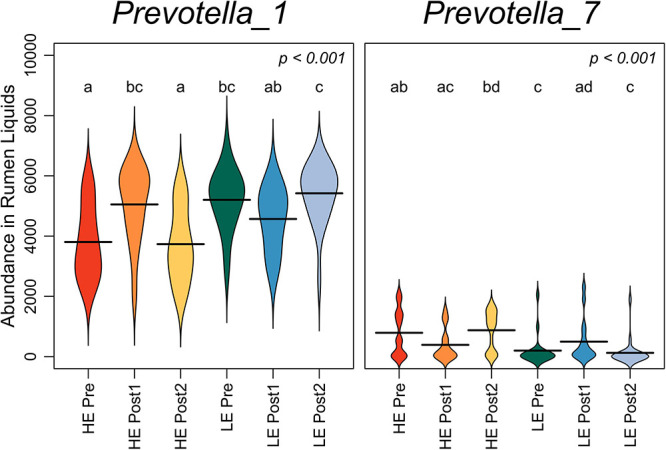
Beanplots expressing abundance of normalized bacterial reads classifying to genera *Prevotella_1* and *Prevotella_7* in liquid-derived rumen samples by time within the study and initial host efficiency. Shared letters indicate no difference in read abundance (*p* > 0.05).

The summed abundance of all OTUs classified to genera *Prevotella_1* and *Prevotella_7* was assessed over the time course in rumen liquids (Figure 3). Pre-exchange, *Prevotella_1* was more abundant in LE cows, while *Prevotella_7* was more abundant in HE cows (*P* < 0.05). *Prevotella_1* increased in HE cows in the Post1 period, then returned to pre-exchange abundance. Conversely, *Prevotella_7* increased in LE cows in the Post1 period before returning to pre-exchange abundance in Post2.

In liquid phase samples from HE cows, a fungal OTU classified to the genus *Neocallimastix* decreased from the Pre to Post1 period (Table 1, F_OTU 5), and increased significantly and remained greater than pre-exchange abundance in Post2. The inverse was seen in LE liquids: F_OTU 5 increased from Pre to Post1 and was at an intermediate abundance in Post2. In both HE and LE animals for both solid and liquid rumen fractions, an OTU classified to *Wickerhamomyces anomalus* decreased sharply in Post1 relative to Pre and Post2 (F_OTU 7). Our fungal sequencing was also able to detect the presence of a known silage spoilage organism, *Penicillium roqueforti* (F_OTU 11), in HE solids and in LE liquids and solids. It was present at very low abundance in Pre and Post1, then increased sharply in Post2.

### Phenotypic Correlations

**FIGURE 4:**
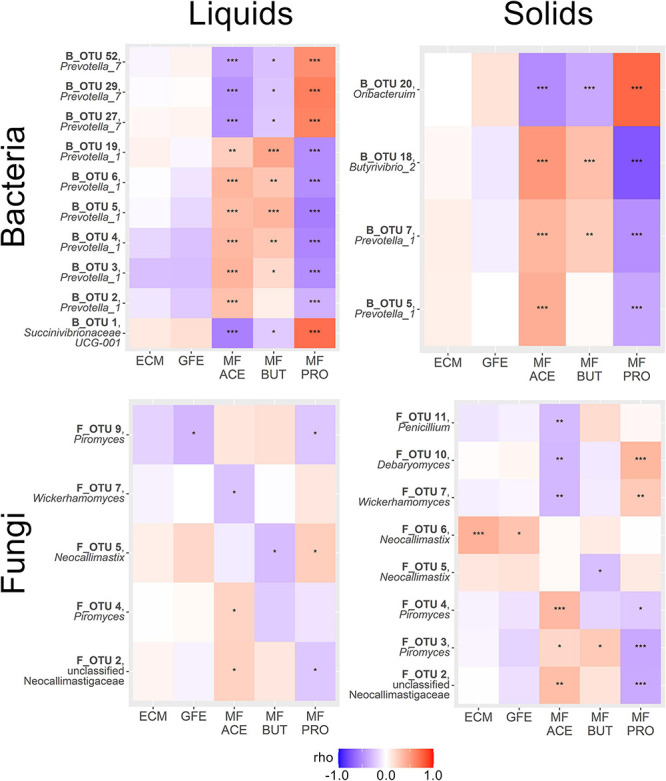
Heatmaps summarizing correlations between SIMPER-implicated OTUs and phenotypic variables of interest within amplicon and sample type. Variables are energy corrected milk (ECM), gross feed efficiency (GFE), and molar fraction acetate (MF ACE), propionate (MF PRO) and butyrate (MF BUT). Genera displayed beneath OTU names. Color scale reflects strength and direction of correlation (Spearman’s ρ statistic), and asterisks within the heatmap indicate statistical significance (*P < 0.05, **P < 0.01, ***P < 0.001).

We then performed a correlation analysis of our SIMPER-implicated OTUs with a number of phenotypic variables. Two production-associated variables (ECM and GFE) and the molar fractions of the three most abundant ruminal VFAs (acetate, propionate, and butyrate) were considered. The results of this correlation analysis are summarized in Figure 4.

Bacterial OTUs correlated to production metrics tended to be weak and non-significant, except for a trend toward a negative relationship between B_OTU 3 (*Prevotella_1*) and both ECM and GFE, and also between B_OTU 2 (*Prevotella_1*) and B_OTU 4 (*Prevotella_1*) with GFE in rumen liquids (*P* < 0.001). Significant and relatively stronger correlations were observed between bacterial OTUs and the relative abundance of specific VFAs. Several bacterial OTUs had highly significant positive correlations to the molar fraction of acetate in rumen liquids (B_OTU 2: *Prevotella_1*, B_OTU 3: *Prevotella_1*, B_OTU 4: *Prevotella_1*, B_OTU 5: *Prevotella_1*, B_OTU 6: *Prevotella_1*, and B_OTU 19: *Prevotella_1*), and several had significant negative correlations to acetate (B_OTU 1: *Succinivibrionaceae UCG-001*, B_OTU 27: *Prevotella_7*, B_OTU 29: *Prevotella_7*, and B_OTU 52: *Prevotella_7*). Similarly, a number of bacterial OTUs were significantly positively (B_OTU 3: *Prevotella_1*, B_OTU 4: *Prevotella_1*, B_OTU 5: *Prevotella_1*, B_OTU 6: *Prevotella_1*, and B_OTU 19: *Prevotella_1*) and negatively (B_OTU 1: *Succinivibrionaceae UCG-001*, B_OTU 27: *Prevotella_7*, B_OTU 29: *Prevotella_7*, and B_OTU 52: *Prevotella_7*) correlated to butyrate abundance in rumen liquids. Propionate tended to have the strongest correlation coefficients in rumen liquids, and followed the exact inverse pattern of acetate in terms of direction of correlation to OTUs the interrogated (positive: B_OTU 1: *Succinivibrionaceae UCG-001*, B_OTU 27: *Prevotella_7*, B_OTU 29: *Prevotella_7*, B_OTU 52: *Prevotella_7*; negative: B_OTU 2: *Prevotella_1*, B_OTU 3: *Prevotella_1*, B_OTU 4: *Prevotella_1*, B_OTU 5: *Prevotella_1*, B_OTU 6: *Prevotella_1*, and B_OTU 19: *Prevotella_1*). No significant correlation was seen between ECM or GFE and the SIMPER-implicated OTUs in rumen solids (*P* > 0.05). Acetate was significantly positively correlated with B_OTU 5 (*Prevotella_1*), B_OTU 7 (*Prevotella_1*), B_OTU 18 (*Butyrivibrio_2*), and negatively correlated with B_OTU 20 (*Oribacterium*) in rumen solids. Butyrate had a positive relationship with B_OTU 7 (*Succinivibrionaceae UCG-002*) and B_OTU 18 (*Butyrivibrio_2*) and a negative relationship with B_OTU 20 (*Oribacterium*). Conversely, propionate was negatively correlated with B_OTU 5 (*Prevotella_1*), B_OTU 7 (*Succinivibrionaceae UCG-002*), and B_OTU 18 (*Butyrivibrio_2*) and strongly positively correlated with B_OTU 20 (*Oribacterium*).

Overall, correlations between the production metrics and the abundance of SIMPER-implicated fungal OTUs were weaker. In rumen liquids, F_OTU 9 (*Piromyces*) was weakly but significantly negatively correlated with GFE. Acetate was positively correlated to F_OTU 2 (unclassified Neocallimastigaceae) and F_OTU 4 (*Piromyces*), and negatively correlated to F_OTU 7 (*Wickerhamomyces anomalus*) in rumen liquids. Negative correlation was observed between butyrate abundance and F_OTU 4 (*Piromyces*) and F_OTU 5 (*Neocallimastix*) in rumen liquids. F_OTU 5 (*Neocallimastix*) was positively correlated to propionate abundance in rumen solids, and F_OTU 2 (unclassified Neocallimastigaceae) and F_OTU 9 (*Piromyces*) were negatively correlated. In rumen solids, F_OTU 6 (*Neocallimastix*) showed a strong positive correlation with ECM, and was the strongest correlation seen between any SIMPER-implicated OTU (bacterial or fungal) and a production metric. F_OTU 6 (*Neocallimastix*) was also positively correlated with GFE in these samples. No other fungal OTUs showed significant correlations with production metrics in rumen solids. In solid samples, acetate was positively correlated with F_OTU 4 (*Piromyces*), F_OTU 5 (*Neocallimastix*), and F_OTU 6 (*Neocallimastix*) and negatively correlated with F_OTU 7 (*Wickerhamomyces anomalus*), F_OTU 10 (*Debaryomyces prosopidis*), and F_OTU 11 (*Penicillium roqueforti*). Butyrate had a positive correlation with F_OTU 3 (*Piromyces*), and no other significant correlations. F_OTU 2 (unclassified Neocallimastigaceae), F_OTU 3 (*Piromyces*), and F_OTU 4 (*Piromyces*) were negatively correlated with propionate abundance in rumen solids, and F_OTU 7 (*Wickerhamomyces anomalus*) and F_OTU 10 (*Debaryomyces prosopidis*) were negatively correlated.

### Linear Discriminant Analysis

**FIGURE 5:**
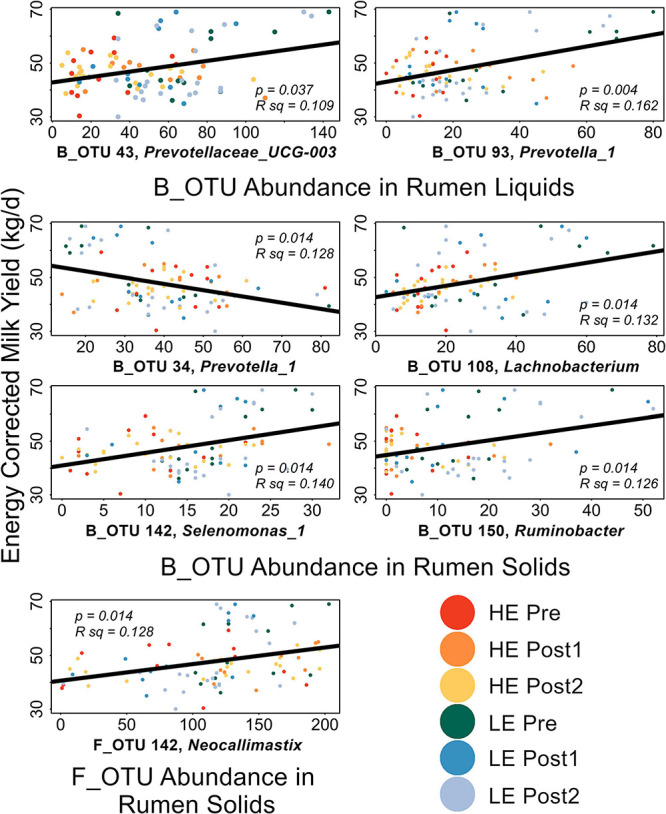
Scatterplots of LEfSe-implicated OTUs with significant correlations to production metrics. FDR-corrected *p*-values and the *R*^2^ values for the linear mode (black) are expressed on each plot. Points are color coded by initial efficiency of host and time within trial. Genus-level classification of the OTUs are as follows: B_OTU 43: *Prevotellaceae_UCG-003*; B_OTU 93: *Prevotella_1*; B_OTU 34: *Prevotella_1*; B_OTU 108: *Lachnobacterium*; B_OTU 150: *Ruminobacter*; B_OTU 142: *Selenomonas_1*; F_OTU 6: *Neocallimastix*.

Linear discriminant analysis effect size implicated several OTUs as diagnostic of HE or LE rumen solids and liquids in the Pre-exchange samples. Implicated OTUs and their effect size are shown in Supplementary Figures 3–6. Within sample type and domain, all LEfSe-implicated OTUs were individually correlated to ECM and GFE (Liquids: 51 B_OTUs, 4 F_OTUs; Solids: 93 B_OTUs, 5 F_OTUs, Supplementary Table 2). Significant correlations are shown in Figure 5. GFE did not show significant correlations with any of the LEfSe-implicated OTUs. In rumen liquids, B_OTU 43 (*Prevotella_UCG3*) and B_OTU 93 (*Prevotella_1*) were significantly positively correlated with ECM. In rumen solids, B_OTU 108 (*Lachnobacterium*), B_OTU 150 (*Ruminobacter*), B_OTU 142 (*Selenomonas_1*), and F_OTU 6 (*Neocallimastix*) were positively correlated with ECM; B_OTU 34 (*Prevotella_1*) was significantly negatively correlated with ECM.

## Discussion

Manipulation of the rumen microbial community is a promising approach for improving MPE (Jami et al., 2014; Bickhart and Weimer, 2017; Weimer et al., 2017). Our previous work demonstrated the ability to alter MPE though wholesale exchange of ruminal contents, but also underscored the resistance of the mature rumen microbiota to long-term perturbation (Weimer et al., 2017). The mechanism behind the re-establishment of the native microbiota following the exchange is not known, but it is likely a confluence of several factors, which may include re-seeding of the lumen by the epimural community; differences in physical factors such as rumen retention, meal frequency, and fluid intake; bioactive compounds in saliva; and host modulation of ruminal VFA profiles. The goal of this work was to more thoroughly investigate rumen microbial community recovery with an emphasis on the ruminal fungal community, and to identify specific microbial taxa that may have contributed to the observed shift in host efficiency.

Weimer et al. (2017) established that ruminal bacterial diversity, richness, and community structure tended to shift from the pre-exchange baseline to reflect the donor community in the Post1 period, then returned to a more similar pre-exchange community in Post2. Here, we reprocessed the original bacterial sequence data using updated analysis methodologies and found that the conclusions drawn from alpha and beta diversity analysis, in response to the exchange, are upheld in this study.

The rumen fungal community, although functionally important in fiber degradation, did not differ pre-exchange between HE and LE hosts by either alpha or beta diversity metrics in our analysis. This contradicts our initial hypothesis and was unexpected given the known importance of anaerobic fungi in improving the digestibility of lignocellulosic feed in the rumen (Russell and Hespell, 1981; Tripathi et al., 2007; Sehgal et al., 2008; Gao et al., 2013; Puniya et al., 2015). Given this lack of contrast pre-exchange, it is perhaps not unexpected that changes over the time course were not observed. As such, while the ruminal bacterial community, as a whole, has been demonstrated to correlate to efficiency metrics in dairy cattle (Jewell et al., 2015; Shabat et al., 2016), the contributions of the ruminal fungal community to efficiency phenotypes appears to be through the action of individual influential taxa, rather than through more complex community-scale function. This community-scale similarity between hosts of differing efficiency status may indicate that ruminal fungi play a narrower role *in vivo* than previously thought. This supports the widely held assumption that the primary function of the anaerobic fungi is physical disruption of fibrous tissues in the earliest stages of feed particle colonization. In later colonization, slow-growing fungi are thought to be outcompeted by fiber-adherent bacteria, which would likely have a greater impact on the pool of metabolites available to the host, and therefore have a greater impact on efficiency metrics.

Our analysis of network connectivity quantified by degree-centrality revealed that bacterial communities were more disturbed by the exchange than fungal communities. The degree-centrality of a node in a correlation network is calculated as the number of edges connecting to the node. In our analysis, this represents the number of strong positive correlations a given OTU has to other bacterial and fungal OTUs. In HE samples, both liquid- and solid-associated bacterial communities saw a decrease in average degree-centrality in Post1 relative to Pre, and a recovery in Post2 (though only partially in the case of HE solids). In contrast, LE bacterial communities had less average degree connectivity at the outset and did not recover after the exchange. Generally, it appears that HE communities are more resilient and are more able than the LE communities to recover complex network interactions following a major disturbance. This indicates that the HE microbial community may display greater elasticity and resilience in the face of perturbation, which may underlie the relatively lower bacterial community diversity which has been reported in HE cows (Shabat et al., 2016; Weimer et al., 2017).

This finding points to the potential for establishing exogenous microbial communities in historically LE cows: if LE communities have inherently lower resilience, then it may be possible to introduce long-term, high-resilience HE communities. We note that the exchange protocol used in this study was insufficient to achieve a new stable state in LE cows despite this disparity, and further work should focus on identifying and overcoming barriers to exogenous community introduction. This may include a greater understanding of the influence of both host immunity and genetics on ruminal microbial community maintenance, a consideration of the metabolic capacities of the ruminal microbiota in HE and LE cows, and methodological changes that may aid exogenous community establishment (i.e., rinsing the rumen prior to introducing the new community or intervening early in life prior to the establishment of the adult ruminal microbial community). Additionally, fungal communities tended to have lower degree connectivity than bacterial communities, irrespective of host efficiency status or study phase, which suggests that fungi do not exert a strong influence on efficiency at the community-level. This reinforces the theory that fungi are not major contributors to the pool of metabolites that serve as milk precursors (Russell and Hespell, 1981).

Many of the bacterial OTUs that were found to change over the time course in either LE or HE cows were classified to the genus *Prevotella*. This observation agrees with Jewell et al. (2015) who showed that some members of this genus are strongly correlated (either positively or negatively) to feed efficiency. Recent updates to the SILVA taxonomic classification database allowed for a more thorough taxonomic division of the *Prevotella* based on sequence identity. *Prevotella_1*, which was more abundant in LE cows in the Pre period and was transferred to HE cows in the Post1 period, contains the type species *Prevotella melaninogenica* and the rumen-derived isolate *P. ruminicola* (Henderson et al., 2019). In the Global Rumen Census dataset (Henderson et al., 2019), which was used to resolve these taxa, *Prevotella_1* accounted for approximately 18% of all reads and was present in 100% of the samples. *Prevotella_7* was much less abundant, with an average of 1% of reads and a prevalence of ∼68%. BLAST analysis of the representative sequences of the two SIMPER-implicated OTUs classifying to *Prevotella_7* revealed that they do not have high sequence similarity (>97%) with any cultured isolates of *Prevotella.* The *Prevotella_1* are better characterized, and three of these OTUs have >97% sequence identity with isolates of *P. ruminicola* (B_OTU 2, B_OTU 3, and B_OTU 5), but the rest have below species-level sequence identity with cultured isolates. Because these genus designations were created based on sequence identity, rather than genomic or phenotypic analysis, very little is known about the variation in metabolism that may impact precursors available to the host. *Prevotella* are generally thought to be major producers of propionate and can utilize a diverse range of substrates (Krause et al., 2003), which is reflected in the strong positive correlations between OTUs classified to *Prevotella_7* and the molar fraction of propionate in the rumen fluid in this study. However, our data also shows a negative relationship between OTUs classified to *Prevotella_1* and the molar fraction of propionate, indicating that more research is needed to determine the specific role of members of this genus in the rumen community. The high prevalence of *Prevotella_1* as a member of highly influential nodes in our network analysis implies that this group may play a role in maintenance and recovery of microbial community structures in the rumen.

F_OTU 3 is a member of the genus *Piromyces* and was found to be highly central to pre-exchange networks regardless of efficiency status or sample type. This OTU accounted for 10% of total fungal reads overall (range: 0–42%). This genus, like others in the Neocallimastigomycota, is known to host a large suite of cellulolytic and hemicellulolytic species (Gruninger et al., 2014). However, the large amount of functional redundancy among the rumen anaerobic fungi makes it difficult to determine how this specific OTU might be exerting influence over the larger microbial community network (Gruninger et al., 2018).

The only fungal OTU with a relatively strong, significant positive correlation to any production metric was F_OTU 6, which is classified to the genus *Neocallimastix* in the phylum Neocallimastigomycota and accounted for 4% of fungal reads (range: 0–8.3%). Members of this phylum are obligate anaerobic fungi that are common in the gastrointestinal tracts of herbivores (Akin et al., 1988; Akin and Borneman, 1990; Lee et al., 2000). Cultured representatives of the *Neocallimastix* ferment sugars to lactate, ethanol, formate, and hydrogen (Lowe et al., 1987). In the rumen, they are among a number of anaerobic fungi whose fermentation of cellulose and hemicellulose are crucial to exposing plant surface area to allow bacterial adherence to plant fiber (Akin and Borneman, 1990). In one study, a culture of *Neocallimastix* fed to buffalo calves led to an increase in feed efficiency, which was attributed to improved fermentation of feed (Sehgal et al., 2008). These fungi are difficult to isolate in the lab, which confounds detailed study of metabolism and microbe-microbe interactions. The representative sequence for F_OTU 6 has high sequence identity with *Neocallimastix lanati*, a recent sheep fecal isolate (99.4% identity, JGI MycoCosm BLAST)^2^. This isolate is a promising candidate for probiotic development due to its ability to grow quickly on defined media. Future work assessing the use of *N. lanati* as a probiotic for increasing milk production and feed efficiency should consider the community-level factors that may help this species to establish and be maintained in the rumen.

It is important to note that the fungal primers used in this study were general primers, as opposed to primers specific to rumen anaerobic fungi in the phylum Neocallimastigomycota. As such, our community analysis included organisms which originate in the diet and do not have a known role in feed degradation in the rumen, such as *Penicillium roqueforti* and *Wickerhamomyces anomalus*. In doing so, this work captures the impact of the exchange protocol on the whole fungal community, including but not limited to those members of the community known to be fibrolytic. However, the inclusion of feed-derived fungal taxa in the analysis may have limited our ability to detect differences in functionally important taxa. Future work could include fungal community sequencing with Neocallimastigomycota-specific primers to determine if focusing on this subset of the community might reveal some interesting contrasts.

In this study, we demonstrate that changes in MPE that result from near-total whole rumen contents exchange in dairy cows is driven primarily by the ruminal bacterial community. Surprisingly, we found that the ruminal fungal community did not differ significantly between hosts of disparate historic MPE, indicating that they were not markedly impacted by the exchange protocol. This supports the hypothesis that the primary role of rumen fungi is in physical disruption of feed particles rather than large and impactful contributions to the pool of metabolites that impact downstream production. Two important exceptions are a specific OTU of *Neocallimastix*, which appears to have a positive impact on MPE and whose recent isolation will allow closer study of its unique role in rumen function, and one OTU of *Piromyces* that appears to exert an outsized influence on microbial community networks in the rumen. Future work in whole-rumen probiotics to improve MPE should focus primarily on the bacterial community with particular attention to the bacterial genera *Prevotella_1* and *Prevotella_7* and the fungal genera *Neocallimastix and Piromyces*.

## Data Availability Statement

The datasets presented in this study can be found in online repositories. The names of the repository/repositories and accession number(s) can be found below: https://www.ncbi.nlm.nih.gov/bioproject/PRJNA329260/, PRJNA329260 and https://www.ncbi.nlm.nih.gov/bioproject/PRJNA695353/, PRJNA695353.

## Ethics Statement

The animal study was reviewed and approved by the College of Agricultural and Life Sciences Animal Care and Use Committee, University of Wisconsin-Madison.

## Author Contributions

GS conceived and designed the experiments. MC, CD, and GS performed the experiments. MC analyzed the data and wrote the original draft. MC and GS acquired the funding supporting this study. All authors reviewed and approved the final manuscript.

## Conflict of Interest

The authors declare that the research was conducted in the absence of any commercial or financial relationships that could be construed as a potential conflict of interest.

## Footnotes

1 http://huttenhower.sph.harvard.edu/galaxy

2 https://mycocosm.jgi.doe.gov/Neolan1/Neolan1.home.html
