# Supplementary material for: Assessing the Response of Ruminal Bacterial and Fungal Microbiota to Whole-Rumen Contents Exchange in Dairy Cows
Source: Front Microbiol. 2021 Jun 1;12:665776. doi: 10.3389/fmicb.2021.665776 (PMC8203821; doi:10.3389/fmicb.2021.665776)
Supplement: Supplementary file 4 [file Data_Sheet_2.DOCX]

**Suen Lab Mothur SOPs**

**Bacterial SOP**

make.contigs(file=example.files)

summary.seqs(fasta=current)

screen.seqs(fasta=current, group=current, maxambig=0, maxlength=300, maxhomop=8)

unique.seqs(fasta=current)

count.seqs(name=current, group= current)

summary.seqs(count=current, fasta= current)

align.seqs(fasta=current, reference=silva.nr_v132.align)

summary.seqs(fasta=current, count=current)

screen.seqs(fasta=current, count=current, summary=current, start=13862, end=23444)

summary.seqs(fasta=current, count=current)

filter.seqs(fasta=current, vertical=T, trump=.)

unique.seqs(fasta=current, count=current)

pre.cluster(fasta= current, count= current, diffs=2)

summary.seqs(fasta=current, count=current)

chimera.uchime(fasta=current, count=current, dereplicate=t)

remove.seqs(fasta= current, count= current, accnos= current)

summary.seqs(fasta=current, count=current)

classify.seqs(fasta=current, count=current, reference=silva.nr_v132.align, taxonomy=silva.nr_v132.tax, cutoff=80)

remove.lineage(fasta=current, count=current, taxonomy=current, taxon=unknown;-Archaea;-Eukaryota;)

summary.seqs(fasta=current, count=current)

split.abund(fasta=current, count=current, cutoff=1)

dist.seqs(fasta= current)

cluster.split(column=current, count= current, method=opti, cutoff=0.03)

make.shared(list=current, count=current, label=0.03)

classify.seqs(fasta=current, count=current, template=silva.nr_v132.align, taxonomy=silva.nr_v132.tax, cutoff=80)

classify.otu(list=current, taxonomy=current, count=current, label=0.03, cutoff=80, basis=otu, probs=F)

summary.single(shared=current, label=0.03, calc=nseqs-sobs-coverage)

normalize.shared(shared=current, norm=10000)

summary.single(shared=current, label=0.03, calc=coverage-nseqs-sobs-chao-shannon)

get.oturep(column=current,list=current, count=current, rename=TRUE, method=abundance, fasta=current)

**Fungal SOP**

make.contigs(file=example.files)

summary.seqs(fasta=current)

screen.seqs(fasta=current, group=current, maxambig=0, maxlength=600, maxhomop=8)

unique.seqs(fasta=current)

count.seqs(name=swap_fungi.trim.contigs.good.names, group=swap_fungi.contigs.good.groups)

summary.seqs(count=current)

pre.cluster(fasta=current, count=current, diffs=4, align=needleman)

summary.seqs(fasta=current, count=current)

chimera.uchime(fasta=current, count=current, dereplicate=t)

remove.seqs(fasta=current, count= current, accnos= current)

summary.seqs(fasta=current, count=current)

classify.seqs(fasta= current, count= current, reference=UNITEv6_sh_99.fasta, taxonomy=UNITEv6_sh_99.tax, cutoff=80)

remove.lineage(fasta=current, count=current, taxonomy=current, taxon=unknown;)

summary.seqs(fasta=current, count=current)

pairwise.seqs(fasta= current, cutoff=0.1)

cluster.split(column=current, count= current, method=opti, cutoff=0.03)

make.shared(list=current, count= current, label=0.03)

classify.seqs(fasta=current, count=current, template=UNITEv6_sh_99.fasta, taxonomy=UNITEv6_sh_99.tax, cutoff=80)

classify.otu(list=current, taxonomy=current, count=current, label=0.03, cutoff=80, basis=otu, probs=F)

summary.single(shared=current, label=0.03, calc=nseqs-sobs-coverage)

normalize.shared(shared=current, norm=2450)

summary.single(shared=current, label=0.03, calc=coverage-nseqs-sobs-chao-shannon)

get.oturep(column= current, count= current, rename=TRUE, method=abundance, fasta= current)
