# Supplementary material for: Assessing the Response of Ruminal Bacterial and Fungal Microbiota to Whole-Rumen Contents Exchange in Dairy Cows
Source: Front Microbiol. 2021 Jun 1;12:665776. doi: 10.3389/fmicb.2021.665776 (PMC8203821; doi:10.3389/fmicb.2021.665776)
Supplement: Supplementary file 7 [file Data_Sheet_5.PDF]

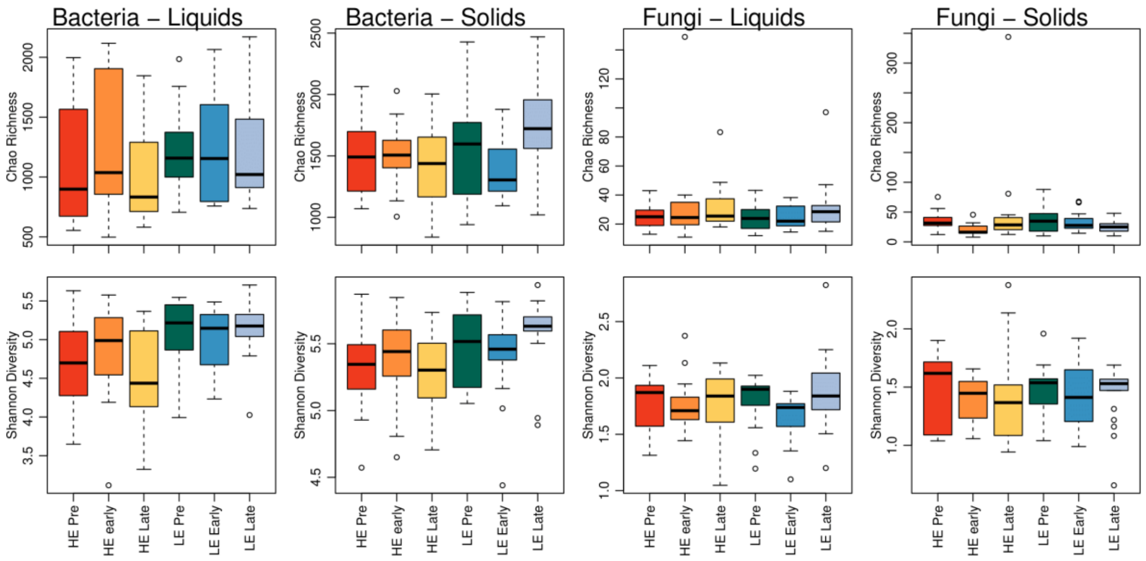

Fig. S1. Boxplots expressing Chao1 richness and Shannon's diversity index over time with HE and LE animals, separated by rumen phase and microbial domain.

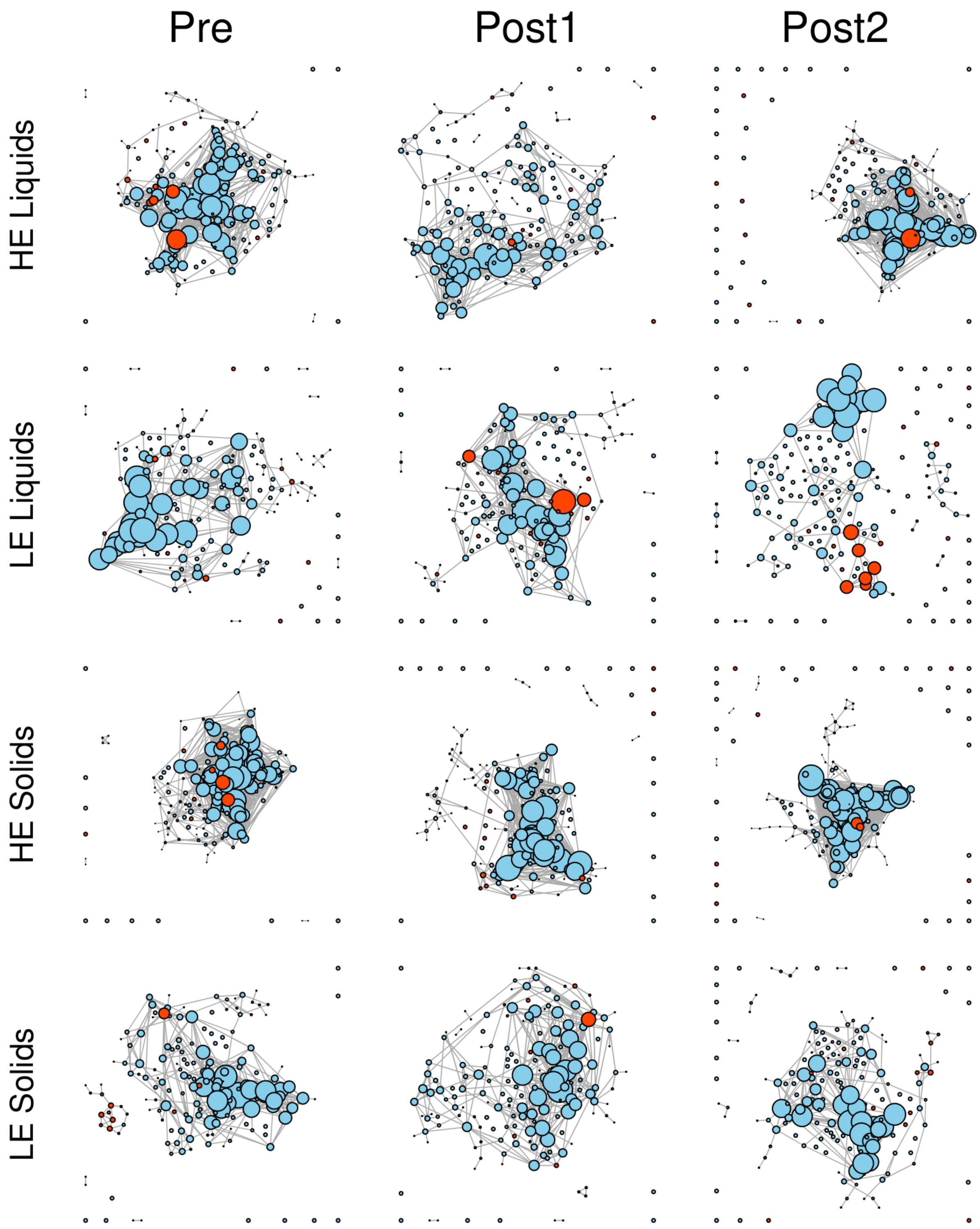

Fig. S2. Correlation networks of rumen communities, separated by initial host efficiency, rumen phase, and time period. Blue nodes represent bacterial OTUs, and orange nodes represent fungal OTUs. Nodes are sized proportional to the degree centrality of the OTU. Strong correlations are visualized as gray edges.

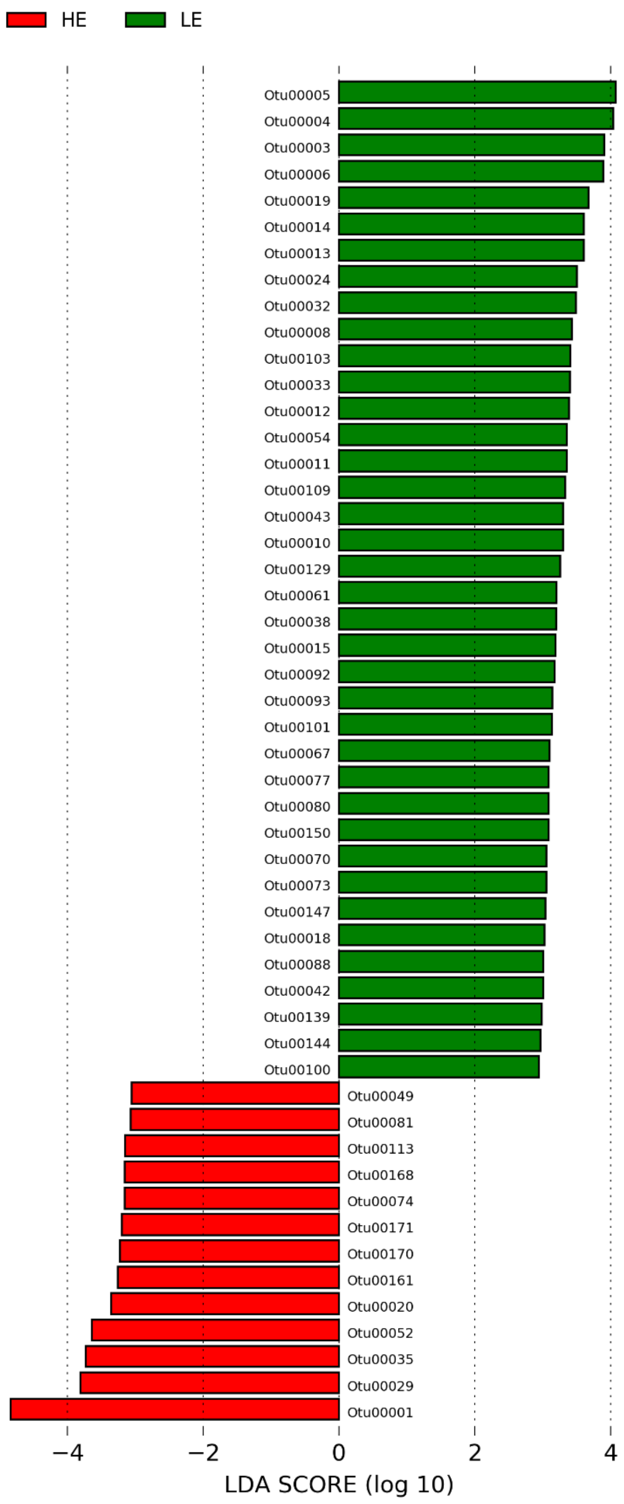

Fig. S3. Linear discriminant analysis scores of LEfSe-implicated bacterial OTUs shown to differentiate between HE and LE rumen liquid samples in the Pre period. OTUs diagnostic of HE samples are shown in red (left), and those diagnostic of LE samples are shown in green (right).

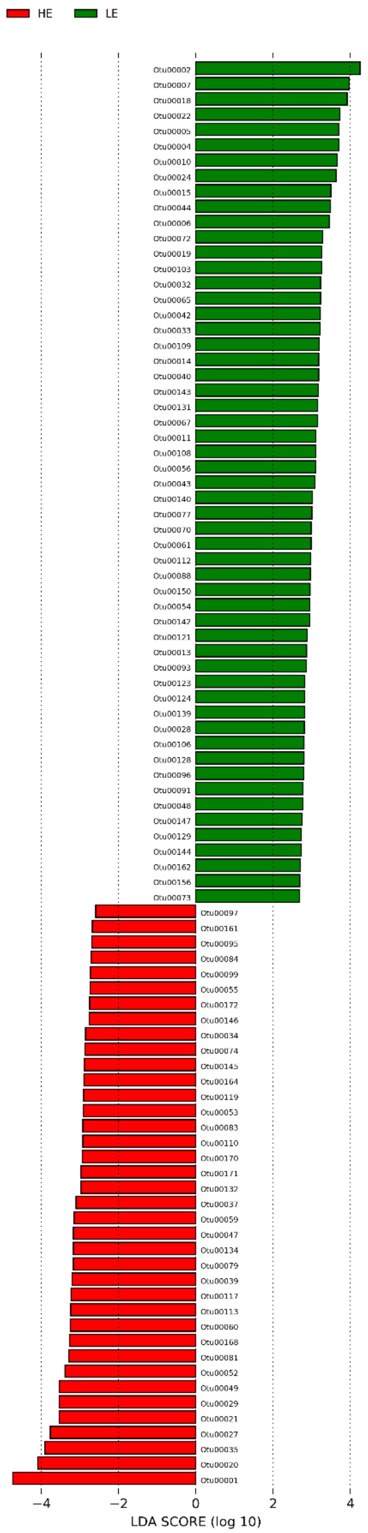

Fig. S4. Linear discriminant analysis scores of LEfSe-implicated bacterial OTUs shown to differentiate between HE and LE rumen solid samples in the Pre period. OTUs diagnostic of HE samples are shown in red (left), and those diagnostic of LE samples are shown in green (right).

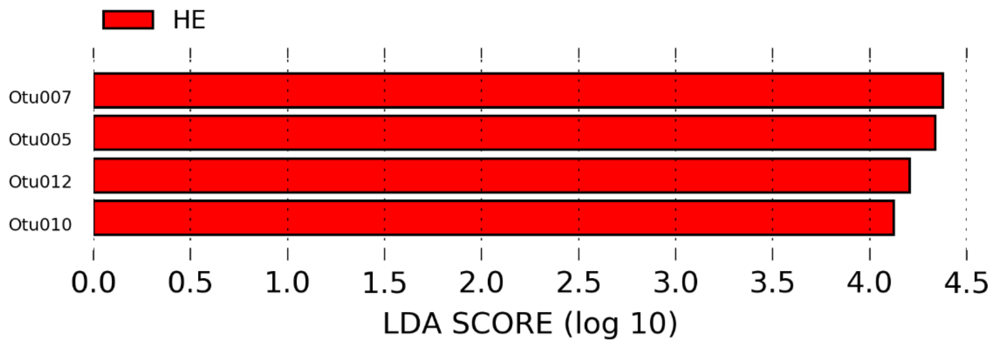

Fig. S5. Linear discriminant analysis scores of LEfSe-implicated fungal OTUs shown to differentiate between HE and LE rumen liquid samples in the Pre period. OTUs diagnostic of HE samples are shown in red.

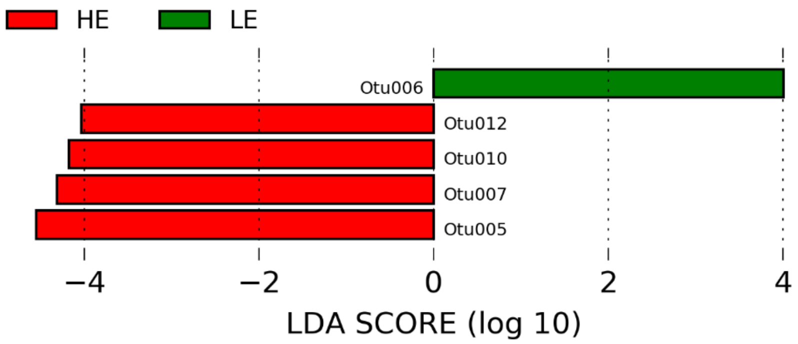

Fig. S6. Linear discriminant analysis scores of LEfSe-implicated fungal OTUs shown to differentiate between HE and LE rumen solid samples in the Pre period. OTUs diagnostic of HE samples are shown in red (left), and those diagnostic of LE samples are shown in green (right).
